# Astrocyte reactivity influences the association of amyloid-β and tau biomarkers in preclinical Alzheimer’s disease

**DOI:** 10.21203/rs.3.rs-2507179/v1

**Published:** 2023-02-01

**Authors:** Tharick Pascoal, Bruna Bellaver, Guilherme Povala, Pamela Ferreira, João Pedro Ferrari-Souza, Douglas Leffa, Firoza Lussier, Andrea Benedet, Nicholas Ashton, Gallen Triana-Baltzerz, Hartmuth Kolbzh, Cèile Tissot, Joseph Therriault, Stijn Servaes, Jenna Stevenson, Nesrine Rahmouni, Oscar Lopez, Dana Tudorascu, Victor Villemagne, Milos Ikonomovic, Serge Gauthier, Eduardo Zimmer, Henrik Zetterberg, Kaj Blennow, Howard Aizenstein, William Klunk, Beth Snitz, Pauline Maki, Rebecca Thurston, Ann Cohen, Mary Ganguli, Thomas Karikari, Pedro Rosa-Neto

**Affiliations:** University of Pittsburgh; University of Pittsburgh; University of Pittsburgh; University of Pittsburgh; Universidade Federal do Rio Grande do Sul; University of Pittsburgh; University of Pittsburgh; Department of Psychiatry and Neurochemistry, Institute of Neuroscience and Physiology, University of Gothenburg, Mölndal, Sweden; Department of Psychiatry and Neurochemistry, Institute of Neuroscience and Physiology, University of Gothenburg, Mölndal, Sweden; Janssen Research and Development; Janssen Research and Development; McGill University; McGill University; McGill University; McGill University; McGill University; Departments of Neurology and Psychiatry, University of Pittsburgh; University of Pittsburgh; University of Pittsburgh; University of Pittsburgh; McGill University; Universidade Federal do Rio Grande do Sul; University of Gothenburg; University of Gothenburg; University of Pittsburgh Medical Center; University of Pittsburgh; University of Pittsburgh; University of Illinois; University of Pittsburgh; University of Pittsburgh; University of Pittsburgh; University of Pittsburgh; McGill University

## Abstract

An unresolved question for the understanding of Alzheimer’s disease (AD) pathophysiology is why a significant percentage of amyloid β (Aβ)-positive cognitively unimpaired (CU) individuals do not develop detectable downstream tau pathology and, consequently, clinical deterioration. *In vitro* evidence suggests that reactive astrocytes are key to unleashing Aβ effects in pathological tau phosphorylation. In a large study (*n*=1,016) across three cohorts, we tested whether astrocyte reactivity modulates the association of Aβ with plasma tau phosphorylation in CU people. We found that Aβ pathology was associated with increased plasma phosphorylated tau levels only in individuals positive for astrocyte reactivity (Ast+). Cross-sectional and longitudinal tau-PET analysis revealed that tau tangles accumulated as a function of Aβ burden only in CU Ast+ individuals with a topographic distribution compatible with early AD. Our findings suggest that increased astrocyte reactivity is an important upstream event linking Aβ burden with initial tau pathology which might have implications for the biological definition of preclinical AD and for selecting individuals for early preventive clinical trials.

Rapid advances in fluid and neuroimaging biomarkers have facilitated the understanding of the dynamic associations between Alzheimer’s disease (AD)-related pathophysiological processes in the living human brain. These biomarker studies suggest that brain accumulation of amyloid-β (Aβ) precedes tau pathology in cognitively unimpaired (CU) individuals^1–3^, which is closely related to the development of cognitive symptoms^4–6^. However, the reasons why Aβ pathology is not associated with AD-related progression in some CU individuals is one of the most pressing questions in the field^7^·^8^. In addition to revealing key biological players associated with disease progression, finding predictive markers of early Aβ-related tau pathology would allow for the identification of CU individuals who are more likely to develop AD even before the first signs of pathological tau, facilitating enrollment in early prevention clinical trials.

The fact that Aβ leads to tau pathology in some individuals, but not in others, suggests the presence of other biological processes capable of triggering the deleterious effects of Aβ in the early disease stages. Postmortem studies show that astrocyte reactivity is a common neuropathological finding and, like cortical Aβ plaques, one of the earliest abnormalities in the AD brain^9–11^. The extent to which astrocyte reactivity contributes to Aβ and tau pathology is not clear. Experimental literature suggests that astrocyte reactivity is critical for triggering Aβ-induced tau phosphorylation^12^ and that the attenuation of astrocyte reactivity mitigates tau pathology^13,14^. Additionally, glial fibrillary acidic protein (GFAP)-positive astrocytes can internalize tau and might contribute to its propagation^15,16^. Furthermore, the release of signaling mediators, including cytokines and caspases, has also been postulated as a mechanism linking reactive astrocytes with tau phosphorylation^12,17–19^.

Clinical studies support that plasma measures of GFAP correlate with its CSF levels, and are increased in CU individuals with AD pathophysiology, representing a robust proxy of astrocyte reactivity in the brains of living individuals^20–22^. Based on this previous literature, we designed a multi-site biomarker study including three cohorts to test the hypothesis that the presence of astrocyte reactivity biomarker abnormality is a key element determining the association of Aβ with early tau phosphorylation and aggregation biomarkers in preclinical AD.

To this end, we investigated 1,016 CU individuals (mean age = 69.6 ± 8.9, CDR = 0) from two research (TRIAD, McGill University, Canada and Pittsburgh, University of Pittsburgh, USA) and one community-based (MYHAT, Pittsburgh, USA) cohort with *in vivo* biomarkers. Individuals were classified as negative (Ast−) or positive (Ast+) for astrocyte reactivity biomarker according to their plasma GFAP levels (see Methods). Demographic and clinical characteristics of participants are summarized in Table 1. Overall, participants classified as Aβ+/Ast+ presented increased plasma p-tau181, p-tau231, and p-tau217 compared to other groups. No differences in Aβ levels were observed between CU Aβ+/Ast− and Aβ+/Ast+ in any cohort. Demographic characteristics of individuals segregated by cohort are presented in **Supplemental Tables 1–3**.

First, we z-scored biomarker levels inside each cohort and applied a robust local weighted regression to model the trajectory of plasma p-tau181, the only p-tau biomarker available in all cohorts, as a function of Aβ burden [plasma or positron emission tomography (PET)] in CU individuals classified as Ast− (*n* = 743) or Ast+ (*n* = 273). Notably, we observed that plasma p-tau181 levels increased as a function of Aβ only in CU Ast+ individuals (Fig.1a) Similarly, linear regression showed a significant association between Aβ burden and plasma p-tau181 in CU Ast+ (β = 0.34, t = 5.37, p < 0.0001; Fig.1b, Supplemental Table 4) but not in CU Ast− (β = 0.04, t = 1.06, p = 0.29; Fig.1 b) individuals. A significant interaction between Ap burden and astrocyte reactivity status on plasma p-tau181 (β = 0.31, t = 4.62, p < 0.0001; Fig.1 b) further supported the presence of astrocyte reactivity was key to determining Aβ effects on tau phosphorylation. Local weighted regression using only continuous values for Aft p-tau181, and GFAP levels confirmed that these results were not influenced by biomarker thresholds (β = 0.10, t = 3.22, p = 0.0013, Fig.1c). Cohen’s *d* analysis revealed that the presence of Aβ+ and Ast+ has a large magnitude of effect on tau phosphorylation (Cohen’s *d* = 0.80), whereas Aβ+ in the absence of Ast+ presented a negligible effect size (Fig.1 d). Voxel-wise analysis confirmed that Aβ levels in brain regions known to present early Aβ plaque accumulation in AD, including the posterior cingulate, precuneus, and insula^23^ associated with plasma p-tau181 only in the presence of astrocyte reactivity (Fig.1e).

Consistently, the stratified analysis within cohorts showed the same results. In the three enrolment sites, plasma p-tau181 levels increased as a function of Aβ burden only in CU Ast+ [Pittsburgh: β= −0.35, t = 3.10, p = 0.003 (Fig.1f), MYHAT: β = −0.20, t = 2.26, p = 0.026 (Fig.1h) and TRIAD: β = 0.57, t = 4.36, p < 0.0001 (Fig.1 j)]. A steeper increase in plasma p-tau181 was observed in the research cohorts (TRIAD and Pittsburgh) compared to the community-based cohort (MYHAT). Similarly, we observed a significant interaction between Aβ burden and astrocyte reactivity status on plasma p-tau181 levels in the Pittsburgh (β = −0.29, t = 2.30, p = 0.022; Fig.1g), MYHAT (β = −0.19, t = 2.07, p = 0.039; Fig.1i) and TRIAD (β = 0.46, t = 2.92, p = 0.004; Fig.1k) cohorts. In a subset of participants from the Pittsburgh and MYHAT cohorts with available Aβ-PET (n = 150), we found the same results with increased plasma p-tau181 as a function of Aβ only in Ast+ (**Supplemental Fig.1**).

We also explored the impact of Ast+ in the associations of Aβ burden with plasma p-tau231 (available for Pittsburgh and TRIAD cohorts, *n* = 502) and p-tau217 (available for the TRIAD cohort, *n* = 136) levels in subsets of individuals that had these markers available. Plasma p-tau231 increased as a function of Aβ only in CU Ast+ individuals (Fig.1l). Additionally, we found a significant association between Aβ and plasma p-tau231 in CU Ast+ (β = 0.36, t = 4.62, p < 0.0001; Fig.1m, **Supplemental Table 5**) but not in CU Ast− individuals (β = 0.10, t = 1.87, p = 0.06). We also observed a significant interaction between Aβ and astrocyte reactivity status on plasma p-tau231 (β = 0.26, t = 2.84, p = 0.004; Fig.1m). Cohen’s *d* analysis suggests that the presence of both Aβ+ and Ast+ also had a strong effect on the levels of p-tau231 (Cohen’s *d*=0.91), whereas pathologies independently did not have a significant effect (Fig.1n). Similarly, plasma p-tau217 presented a steeper increase as a function of Aβ burden in Ast+ compared to Ast− (Fig.1o, Supplemental Table 5). An association between Aβ burden and plasma p-tau217 was observed in Ast− (p = 0.19, t = 3.48, p = 0.0008, Fig.1 p), but with a much larger magnitude in Ast+ (p = 0.74, t = 5.62, p < 0.0001, Fig.1 p) individuals. The stronger association in CU Ast+ individuals was further evidenced by a significant interaction between Aβ burden and astrocyte reactivity status on plasma p-tau217 (p = 0.52, t = 3.61, p = 0.0004, Fig.1 p). The presence of both Aβ+ and Ast+ had the largest effect size on plasma p-tau217 increase (Cohen’s *d* = 1.41; Fig.1 q) compared to p-tau181 and p-tau231. Importantly, a sex effect was observed in the association between Aβ and plasma p-tau epitopes in CU Ast+ individuals in all cohorts, with the association being stronger in men than women (Fig.1r, Supplemental Fig.2). The greater effect of Aβ burden on tau phosphorylation in the presence of Ast+ in men than in women may prove to play a role in the larger magnitude of effect of anti-Aβ therapies in man^24^. Finally, the presence of astrocyte reactivity did not impact the association between Aβ burden and NfL levels in any of the three cohorts (**Supplemental Table 6**), supporting that astrocyte reactivity unleashes Aβ effects on early tau pathology.

We used PET imaging available in the TRIAD cohort to determine the topographic localization of p-tau protein aggregates in the form of tangles (*n* = 147). Tau-PET deposition occurred as a function of Aβ burden only in CU Ast+ and in regions expected to present the earliest tau deposition (Fig.2a), affecting 100% and 62% of the extension of the Braak I and II regions, respectively (Fig.2b). As expected, in later Braak regions tau-PET did not increase as a function of Aβ in either group (Fig.2b). Finally, we investigated the link of baseline Aβ and astrocyte reactivity status with future tau-PET burden (*n* = 71; mean follow-up = 2.3 years; **Supplemental Table 7**). We observed that the annual rate of tau-PET accumulation was higher in the CU Ast+ group (Fig.2c) and predicted by baseline Aβ burden only in CU Ast+ (Fig.2d). Interestingly, while the baseline association was confined to the mesial temporal cortex, the longitudinal tau-PET accumulation as a function of Aβ/Ast presented initial tau spread over the neocortex in Braak III-IV regions (Fig.2e), further supporting the notion that these individuals are following a tau accumulation pathway consistent with AD progression^25^.

In summary, we provide biomarker evidence across multiple cohorts that increased astrocyte reactivity, measured by a plasma GFAP assay, plays a key role in the association of Aβ with early tau pathology in preclinical AD. The fact that the presence of abnormal astrocyte reactivity potentiates Aβ-triggered tau pathology may prove to favor the inclusion of astrocyte reactivity biomarkers in the biomarker modeling^1^ and biological definitions^26^ of AD. The strengths of our study include large sample size and the use of well-characterized research and population-based cohorts. While our cohort represents significant socioeconomic diversity, the main limitation is that our cohorts are composed mainly of White participants, which limits the generalizability of our findings to a more diverse world population. As biomarkers are naturally continuous, dichotomizing thresholds are invariably subject to conceptual and analytical idiosyncrasies and may change depending on the method used.

Furthermore, our findings support recent observations suggesting that plasma p-tau is a state marker of Aβ in preclinical AD^3^, but also add that this occurs mainly with the concomitant presence of astrocyte reactivity biomarker abnormality. As preventive clinical trials have increasingly focused on individuals in the earliest preclinical phases of AD, our results highlight that the selection of CU individuals Aβ+/Ast+ without overt p-tau abnormality may offer a time window very early in the disease process but with increased risk of AD-related progression. Finally, based on our results, we may speculate that a combination of drugs targeting both Aβ and astrocyte activation can potentiate the prevention of early tau pathology in preclinical AD.

## Methods

### Study population

This study included participants from three centers. The Translational Biomarkers in Aging and Dementia (TRIAD) cohort (Montreal, Canada, https://triad.tnl-mcgill.com) comprised participants with a detailed clinical and cognitive assessment. Exclusion criteria included inability to speak English or French, inadequate visual and auditory capacities for neuropsychologic assessment, active substance abuse, major surgery, recent head trauma, medical contraindication for positron emission tomography (PET) or magnetic resonance imaging (MRI), currently being enrolled in other studies, and neurological, psychiatric, or systemic comorbidities that were not adequately treated with a stable medication regimen. CU individuals had a Clinical Dementia Rating (CDR) = 0 and no objective cognitive impairment. The study was approved by the Douglas Mental Health University Institute Research Ethics Board and Montreal Neurological Institute PET working committee.

The Monongahela-Youghiogheny Healthy Aging Team (MYHAT) is an ongoing population-based study cohort drawn from a Rust Belt region of southwestern Pennsylvania, USA^27^. Participants were selected by age-stratified random sampling from the publicly available voter registration lists over two time periods: 2006–2008 and 2016–2019. Inclusion criteria at study entry included: 1) 65+ years old, 2) living in a designated town, 3) not residing in long-term care settings, 4) having sufficient hearing and vision to complete neuropsychological testing, and 5) having decisional capacity. CU individuals had CDR = 0. All study procedures were approved by the University of Pittsburgh Institutional Review Board and all participants provided written informed consent.

The Pittsburgh cohort is composed of research volunteers from four studies conducted at the University of Pittsburgh: the Heart Strategies Concentrating on Risk Evaluation (Heart SCORE) parent study^28^, the Human Connectome Project (HCP)^29^, the Normal Aging study^30^, and the MsBrain^31^. CU individuals were classified using either CDR = 0 or MoCA > 25 (for the MsBrain study). Individuals were selected according to cognitive status and plasma biomarker availability. Details of each cohort recruitment are reported in the **Supplemental Table 8**.

### Plasma Biomarkers

For Pittsburgh and TRIAD cohorts, plasma biomarkers (except for plasma p-tau217) were measured using Single molecule array (Simoa) methods on an HD-X instrument (Quanterix, Billerica, MA, USA), at the Clinical Neurochemistry Laboratory at the University of Gothenburg, Sweden. Plasma Aβ42, Aβ40, GFAP and NfL were quantified with the Neurology 4-Plex E (#103670) commercial assays from Quanterix (Billerica, MA, USA). Plasma p-tau181^32^ and plasma p-tau231^33^ were measured using an in-house Simoa assay developed at the University of Gothenburg, as previously described. Plasma p-tau217 (available only for TRIAD) was quantified by scientists at Janssen Research & Development^34^. For the MYHAT cohort, plasma biomarkers were measured using Simoa methods on an HD-X instrument (Quanterix, Billerica, MA, USA), at the Department of Psychiatry, University of Pittsburgh School of Medicine, USA. Plasma p-tau181 was measured with the p-tau181 V2 Advantage (#103714) while plasma Aβ42, Aβ40, GFAP and NfL concentrations were measured with the Neurology 4-Plex E (#103670) commercial assays from Quanterix (Billerica, MA, USA). For cohorts with no Aβ-PET available *(i.e.*, MYHAT and the Pittsburgh cohort), Aβ positivity was determined using plasma Aβ42/40 based on the expected 30% of Aβ-positivity in CU individuals^35^. As younger individuals are expected to present less AD-related pathology^36,37^, cutoffs for astrocyte reactivity were generated using plasma GFAP mean of the 15% youngest Aβ-negative individuals plus 2 standard deviations (s.d).

### MRI/PET Biomarkers

For the TRIAD cohort, Aβ-PET was quantified using the tracer [^18^F]AZD4694 and Tau-PET with the tracer [^18^F]MK-6240 in a Siemens High Resolution Research Tomograph. [^18^F]AZD4694 and [^18^F]MK-6240 images were acquired at 40–70 min and 90–110 min post-injection, respectively. Standardized uptake value ratio (SUVR) was calculated using the whole cerebellum gray matter for [^18^F]AZD4694 and inferior cerebellum gray matter [^18^F]MK-6240 as reference. Neocortical [^18^F]AZD4694 SUVR value was estimated from precuneus, prefrontal, orbitofrontal, parietal, temporal, anterior, and posterior cingulate cortices. Individuals with Aβ-PET SUVR > 1.55 were considered Aβ-positive^38^. A subsample of 71 CU individuals had a follow-up [^18^F]MK6240 with a mean of 2.3 year after baseline. Tau-PET Braak stage segmentation was previously described elsewhere^39^. Stages consisted of the following regions: Braak I (transentorhinal), Braak II (entorhinal and hippocampus), Braak III (amygdala, parahippocampal gyrus, fusiform gyrus and lingual gyrus), Braak IV (insula, inferior temporal, lateral temporal, posterior cingulate and inferior parietal), Braak V (orbitofrontal, superior temporal, inferior frontal, cuneus, anterior cingulate, supramarginal gyrus, lateral occipital, precuneus, superior parietal, superior frontal and rostromedial frontal) and Braak VI (paracentral, postcentral, precentral and pericalcarine).

A subset of individuals from MYHAT (*n* = 86) and Pittsburgh (*n* = 64) cohorts that had Aβ-PET available were used in this study. For this individuals Aβ-PET was quantified using [^11^C]PiB PET with data collected in a series of 5 min frames spanning 50–70 min post-injection. SUVR from nine composite regional outcomes were computed (anterior cingulate, posterior cingulate, insula, superior frontal cortex, orbitofrontal cortex, lateral temporal cortex, parietal, precuneus, and ventral striatum). A global [^11^C]PiB retention index was computed by volume-weighted averaging of all nine composite [^11^C]PiB regions. Aβ-PET positivity was defined using a previously stablished cutoff^40^.

### Statistical analysis

Neuroimaging analyses were carried out using the VoxelStats toolbox (https://github.com/sulantha2006/VoxelStats), a MATLAB-based analytical framework that allows for the execution of multimodal voxel-wise neuroimaging analyses^41^. Other statistical analyses were performed using the R Statistical Software Package version 3.5.3. Differences between groups in continuous variables [age, cognitive performance (MMSE or MoCA), biomarkers for Aβ plasma GFAP p-tau epitopes and NfL] were *assessed using analysis of variance (ANOVA) with Tukey correction. Kruskal–Wallis with post-hoc Mann-Whitney U-tests were used for categorical or ordinal variables (sex and *APOE* e4 status). For modeling the trajectories of plasma p-tau epitopes as a function of Aβ burden (plasma Aβ or Ap-PET) we corrected each plasma p-tau epitope value by age and sex. Individuals in the 15^th^ lower percentile for Ap-PET or the 15^th^ highest percentile for plasma Aβ42/40 were used as anchor to z-scores. Then, we applied a robust local weighted regression method (Lowess), using 1000 robustifying iterations, with a smoother span of 0.95. The effect size of group differences was estimated by calculating Cohen’s *d*, in which the dependent variable was the plasma biomarkers corrected for age and sex. For Cohen’s d analysis, the Ast+ group included only individuals positive for astrocyte reactivity but Aβ−. The Aβ+ group included only individuals positive for Aβ but Ast−. The associations between biomarkers were assessed with linear regressions accounting for age and sex. The interaction term Aβ burden x astrocyte reactivity status/or plasma GFAP as a continuous variable was also added to each model. For analysis including all cohort, we included cohort as a covariate to adjust for variability in differences between cohorts. For all linear regression analysis, z-scores were centered on the mean within each cohort and z-scores for plasma Aβ ratio were inverted to pool plasma Aβ and Aβ-PET levels together. Voxel-wise associations between biomarkers were tested using linear regressions accounting for age, sex, and adjusted for multiple comparisons using Random Field Theory (RFT) threshold of p < 0.001^42^. To assess individuals’ percentage of abnormal regions we used composite brain regions corresponding to Braak histopathological stages (PET Braak-like stages I-VI). We measured the annual rate of progression in [^18^F]MK-6240 uptake as the difference between follow-up and baseline uptakes divided by time between scans.

## Figures and Tables

**Figure 1:**
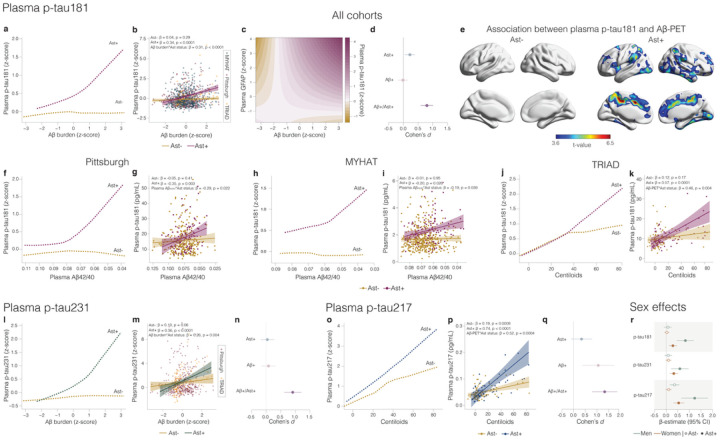
**a,f,h,j** Robust local weighted regressions show that plasma p-tau181 increases as a function of Aβ burden only in the of the presence of astrocyte reactivity (Ast+) in (**a**)all cohorts together (*n*=1,016) as well as in the (**f**) Pittsburgh (n=355),(**h**) MYHAT (n=514) and (**j**) TRIAD (*n*=147) cohorts. **b,g,I,k**Linear regressions revealed an interaction between Aβ burden and astrocyte reactivity status on p-tau181 levels in (**b**) all cohorts, as well as in (**g**) Pittsburgh, (**i**) MYHAT, and (**k**) TRIAD cohorts. **c** Continuous association between Aβ pathology, plasma p-tau181, and plasma GFAP **d,n,q** Cohen’s *d* analysis accounting for age and sex shows the effect sizes of Aβ and Ast on plasma (**d**) p-tau181, (n) p-tau231 and (q) p-tau217. **e** Voxel-wise regressions, corrected for multiple comparison, show that Aβ-PET is associated with plasma p-tau181 only in CU Ast+ in typical AD regions (TRIAD cohort). **l,o** Robust local weighted regression shows that plasma (**l**) p-tau231 and (**o**)p-tau217 increased as a function of Aβ in the presence of astrocyte reactivity. **m,p** Linear regressions revealed an interaction between Aβ burden and reactive astrocyte status on plasma (**m**) p-tau231 and (**p**)p-tau217. **r** β estimates with respective 95% confidence interval of linear regressions showing the effect of sex on the associations of Aβ with plasma p-tau epitopes in Ast− and Ast+.

**Figure 2:**
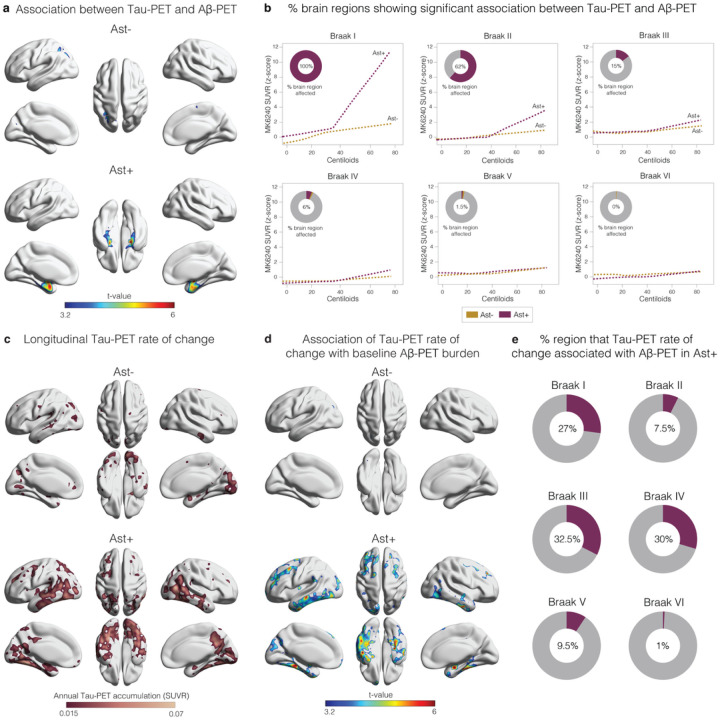
**a** Voxel-wise regression analysis showing the association between Aβ-PET and Tau-PET in individuals classified as negative (Ast−) or positive (Ast+) for astrocyte reactivity (*n*=147). **b**Percentage of the extent of the brain region with significant association (after RFT-correction) between Tau-PET and Aβ-PET in each Braak region. **c**Longitudinal Tau-PET annual rate of change according to astrocyte reactivity status (*n*=71). **d** Association between Tau-PET annual rate of change and baseline Aβ-PET according to astrocyte reactivity status. **e**Percentage of voxels with significant association (after RFT-correction) between Tau-PET annual rate of change and baseline Aβ-PET in each Braak region. Associations were tested using voxel-wise linear regression models corrected for RFT multiple comparison and adjusted by with age and sex.

**Table 1.** Demographics and key characteristics of participants.

	Aβ−/Ast− (*n* = 557)	Aβ−/Ast+ (*n* = 165)	Aβ+/Ast− (*n* = 186)	Aβ+/Ast+ (*n* = 108)
Age, mean (SD)	68.2 (8.6)	72.1 (8.2)^a^	68.6 (7.6)^b^	74.7 (10.6)^a,c^
Sex, *n* (% female)	367 (65.9)	137 (83.0)^a^	122 (65.6)^b^	79 (73.1)
MMSE/MoCA, mean (SD)	28.1 (3.3)/27.5(1.8)	28.1 (3.2)/28.1(1.7)	27.8 (3.2)/26.7(4.1)^b^	27.1 (6.1)/27.2(1.4)
*APOE* e4 (% of carriers)	89 (16.0)	25 (15.2)	33 (17.7)	25 (23.1)
Education, years (SD)	15.0 (2.7)	15.2 (3.1)	14.8 (2.7)	15.0 (2.8)
Aβ burden (z-score)	−0.52 (0.65)	−0.40 (0.57)	1.17 (0.63)^a,b^	1.25 (0.69)^a,b^
Plasma GFAP (z-score)	−0.50 (0.51)	1.20 (0.75)^a^	−0.42 (0.52)^b^	1.48 (0.88)^a^-^b,c^
Plasma p-tau181 (z-score)	−0.12 (0.90)	0.10 (0.82)^a^	−0.14 (0.97)	0.77 (1.89)^a,b,c^
Plasma p-tau231 (z-score)	−0.03 (1.00)	−0.12 (0.82)	−0.04 (0.86)	0.55 (1.26)^a,b,c^
Plasma p-tau217 (z-score)	−0.27 (0.38)	−0.24 (0.38)	0.17 (0.49)	1.12 (1.99)^a,b,c^
Plasma NfL (z-score)	−0.24 (0.62)	0.42 (0.99)^a^	−0.13 (1.22)^b^	0.80 (1.47)^a,b,c^

Abbreviations: Aβ: Amyloid-β; Aβ: Aβ-negative; Aβ+: Aβ-positive; Ast−: reactive astrocyte negative; Ast+: reactive astrocyte positive; *APOE* e4: Apolipoprotein ε4; MMSE: Mini-Mental State Exam; MoCA: Montreal Cognitive Assessment; GFAP: glial fibrillary acidic protein; NfL: neurofilament light chain; p-tau: phosphorylated tau.

a = different from Aβ−Ast−,

b = different from Aβ−/Ast+,

c = different from Aβ+/Ast−.

Missing *APOE* e4: 140 Aβ−/Ast−, 43 A-/Ast+, 45 Aβ+/Ast−, 17 Aβ+/Ast+.Missing NfL: 2 Aβ−/Ast−, 2 Aβ−/Ast+, 1 Aβ+/Ast−. Plasma p-tau231 is available for a subset of participants from TRIAD and Pittsburgh cohorts. Plasma p-tau217 is available for a subset of participants from TRIAD cohort.
